# Accelerating the electrical response of solvent-dispersed imogolite nanotubes through structural organisation†

**DOI:** 10.1039/d0ra01092h

**Published:** 2020-03-05

**Authors:** K. Shikinaka

**Affiliations:** Research Institute for Chemical Process Technology, National Institute of Advanced Industrial Science and Technology (AIST) Sendai 983-8551 Japan kaz.shikinaka@aist.go.jp

## Abstract

Structural organisation of solvent-dispersed imogolite nanotubes accelerated their electrical response, resulting in birefringence variations analogous to a liquid crystal system. Crosslinking, confinement, and helical structuring of the imogolite nanotubes in the solvent led to the cooperative aggregation and dissociation of the dispersed nanotubes, which induced rapid changes in their birefringence.

## Abstract 2

Structural organisation of solvent-dispersed imogolite nanotubes accelerated their electrical response, resulting in birefringence variations analogous to a liquid crystal system.

## Graphical abstract

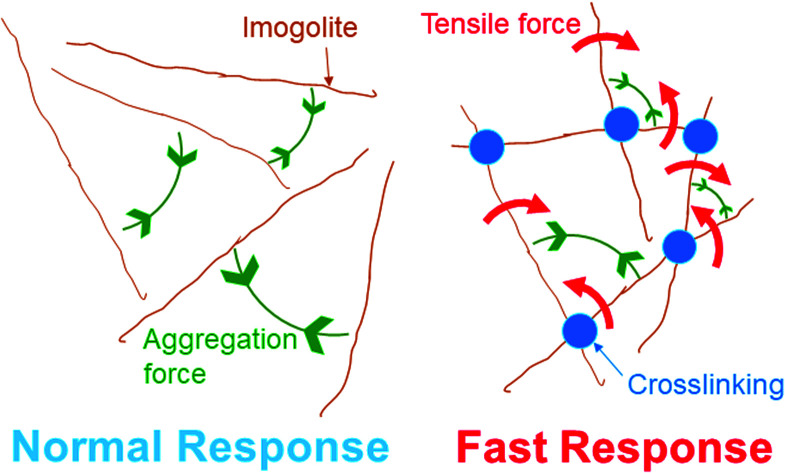

## Introduction

Stimuli-responsive materials with hierarchical structures are essential to realising intelligent, self-tuning, active systems like living creatures. Liquid crystals (LCs) are used to fabricate various stimuli-responsive materials.^1^ The LC systems consisting of inorganic colloids, such as clay minerals, have been energetically studied for the design of soft and wet stimuli-responsive materials.^2–5^ Imogolite (IG) is a single-walled inorganic polymer with a rigid nanocylindrical shape. It is a clay mineral with the composition (HO)_3_Al_2_O_3_MOH (M = Si or Ge).^6,7^ The internal and external diameters of IG nanocylinders are approximately one and two nanometers, respectively. The length of IG nanocylinders can range from several tens of nanometers to several micrometres. IG is a rigid nanotube-like polyelectrolyte with a high aspect ratio,^7^ and it has been used as a constituent of functional soft materials.^8,9^

The outer and inner surfaces of IG nanotubes are covered with proton-capturing Al(OH)_2_ and proton-releasing SiOH groups. Thus, thin IG bundles or monofilaments disperse in acidic and low-ionic-strength polar solvents to yield inorganic colloidal suspensions in water^10^ and ionic liquids. A slightly opaque nanotube dispersion with a maximum concentration of 10% (w/v) was obtained when IG (M = Si) was purified *via* reprecipitation in tetrahydrofuran and sonicated for 4 h in pure water.^11^ A distribution of nanotube lengths was observed, but their average length was decreased from several μm to 131 nm.

The dispersibility of IG in polar solvents and the protonation/deprotonation equilibrium in dilute colloidal dispersions (0.27% w/v) can induce an electrorheological effect. This is a reversible change in the rheological properties of the dispersion due to the aggregation and dissociation of colloidal IG nanotubes upon the application or removal of a direct current (upper 6.0 V mm^−1^).^12^ Applying an alternating current (500 kHz) at 100 V mm^−1^ to a 0.22 wt% aqueous colloidal dispersion of IG (M = Ge) for 10 s induced uniform birefringence, which was due to the assembly of IG nanotubes into LC hexagonal columnar structures that aligned parallel to the direction of the electric field.^13^

## Results and discussion

### Electrical response of IG colloidal dispersion

**Fig. 1:**
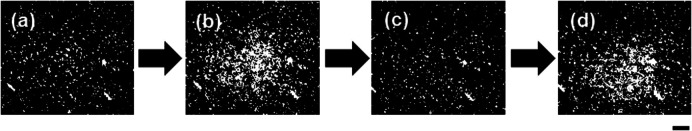
Polarised optical microscope (POM) images of a 6.4% (w/v) aqueous IG dispersion (a) without an applied AC, (b) after application of an AC for 5 min, (c) 5 min after removal of the AC, and (d) after reapplication of an AC for 5 min. The scale bar is equal to 200 μm.

In this paper, I report the acceleration of the electrical response of IG colloidal dispersions through the structuring of IG nanotubes into a network and a helical ordering. The accelerated electrical response caused changes in the birefringence of the dispersions. A 6.4% w/v aqueous IG dispersion (0.16 mol L^−1^ as Al–OH) did not exhibit birefringence (Fig. 1a). The aqueous IG dispersion displayed clear birefringence within 85 s of applying an alternating current (AC) at 3–5 V_p–p_ and 10–1000 kHz (Fig. 1b). The birefringence disappeared within 105 s of removing the current (Fig. 1c). Reapplication of an AC for about 1 min induced the re-emergence of birefringence (Fig. 1d). The IG nanotubes aggregated in the applied electric field due to the disturbance of the electric double layer (EDL) on the IG surface,^12^ which freed domains for the expression of birefringence. The EDL on the IG surface was restored when the electric field was removed, which caused the dissociation of the IG nanotubes and the loss of birefringence.

### Accelerating electrical response of IGs by their gelation

**Fig. 2:**
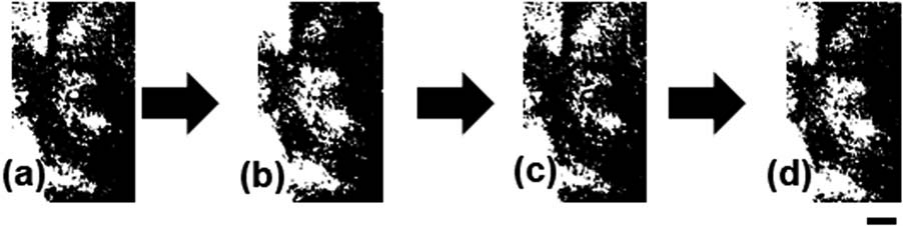
POM images of an IG–maleic acid (IG–MA) gel (a) without an applied AC, (b) after application of an AC for 1 min, (c) 1 min after removal of the AC, and (d) after reapplication of an AC for 1 min. [IG] = [MA] = 0.08 mol L^−1^ with –Al(OH)_2_ and carboxyl groups in a 1 : 1 molar ratio. The scale bar is equal to 200 μm.

When mixed with dicarboxylic acid (DA), –Al(OH)_2_ groups on the IG colloidal nanotubes crosslinked with carboxyl groups^14^ in DA to form a network. Formation of the Al(OH)O^+^H_2_⋯^−^OOC–(CH_2_)_*n*_–COOH (*n* > 1) structure transformed the IG colloidal dispersion into a gel, hereafter referred to as IG–DA gel. Upon the application of a mechanical force such as vibration, the IG–DA gel underwent rapid thixotropic phase changes. These are solid-to-liquid or liquid-to-solid transitions on a sub-second timescale.^15,16^ The thixotropic nature of the IG–DA gel allowed it to be sealed in a narrow cell, and changes in its birefringence could be estimated. An IG gel prepared with maleic acid (MA) exhibited birefringence in the absence of an applied electric field (Fig. 2a). However, the birefringence of the IG–MA gel changed upon the application of an AC (Fig. 2b). As observed with a neat aqueous IG dispersion, changes in its birefringence in response to an applied AC were reversible. This indicated the crosslinks in the IG–MA gel were retained, even when an electric field was applied, and that the IG nanotubes were aggregated (Fig. 2c and d).

**Fig. 3:**
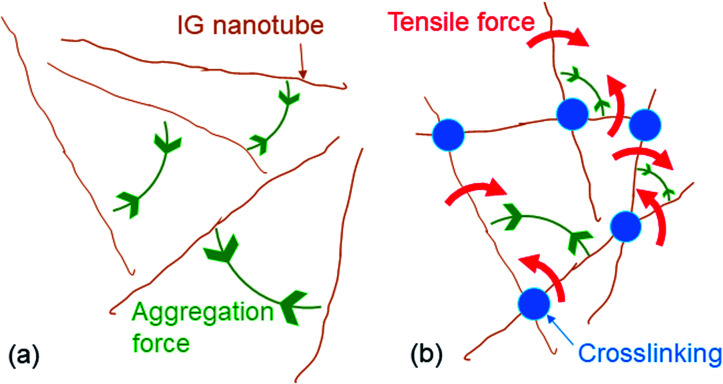
Schematic illustration of IG nanotube aggregation in (a) an IG dispersion and (b) an IG–DA gel.

Gelation of the aqueous IG dispersions accelerated changes in their birefringence in response to the application or removal of an AC (Table 1). The birefringence of the IG–MA gel changed five times faster than that of a neat aqueous IG dispersion after an AC was applied. When the AC was removed, the birefringence of the gel changed 3.2 times faster than that of the neat dispersion. The cooperative aggregation of crosslinked IG nanotubes in an electric field thus caused the birefringence of the IG colloidal dispersions to change more rapidly. This indicated that the aggregation of IG nanotubes (Fig. 3a) within the network placed additional tensile force on adjacent crosslinked IG nanotubes like as IG nanotubes linked in pulled polymer networks,^11^*i.e.*, traction for IG nanotubes in their network is emerged by an external force. This is indicated by the red arrows in Fig. 3b.

**Table 1:** Time required for birefringence (BF) changes after applying or removing AC (5 V_p–p_, 10 kHz). The time was recorded when BF reached a plateau. The samples were then monitored for at least 30 minutes to ensure no additional BF changes occurred. Each sample was placed between the ITO glass slides with a 30 μm of spacer

Sample	Time to BF change after applying AC (s)	Time to BF change after removing AC (s)
Neat IG	85	105
IG + MA	17	32
IG + MaA	16	23
IG + MaA + IL	6.0	4.1

An IG gel was prepared with malic acid (MaA), an analogue of DA with a chiral carbon. The birefringence of the IG–MaA gel changed 1.4 times faster than that of the IG–MA gel after removal of an electric field (Table 1). I previously reported the creation of a one-handed helical architecture on IG colloidal nanotubes that were crosslinked with MaA. The IG–MaA gels exhibited a 4.6 μm pitch band in their flow orientation.^17^ Their helical architecture was expected to induce the IG nanotubes to undergo structural transitions more quickly. This would explain why the birefringence of the IG–MaA gel changed more rapidly than that of the IG–MA gel.

In the narrow cell used to estimate changes in birefringence, it appeared that the accelerated electrical response due to helical ordering of the colloidal IG nanotubes caused the IG–MaA gel to behave like a ferroelectric LC.^18^ Steric hindrance due to the helical pitch of the ferroelectric LC near the cell gap stabilised its surface, which enabled a high-speed electrical response.^19^ The helically ordered IG nanotubes in our LC system had two stable orientations, which induced the LC molecules to undergo rapid structural transitions. High-speed modulation of birefringence on a crossed nicol state in response to the application or removal of an electric field was thus possible. Changes on the birefringence of IG–DA gels in response to an electric field actually became slower as the size of the spacer increased (Table S1†). This demonstrated the important influence of steric hindrance on the speed of the electrical response of IG nanotubes in confined spaces.

**Fig. 4:**
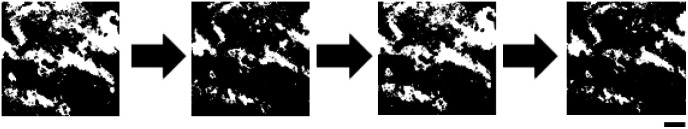
POM images of an IG–MaA gel dispersed in [EmIm][MeSO_3_] (a) without an applied AC, (b) after application of an AC for 10 s, (c) 1 min after removal of the AC, and (d) after reapplication of the AC for 1 min. [IG] = [MaA] = 0.08 mol L^−1^. The scale bar is equal to 200 μm. [EmIm][MeSO_3_] is 1-ethyl-3-methylimidazolium methanesulfonate.

The solvent change of IG–DA gel^20^ also affected its electrical response (Fig. 4). In contrast to pure water, an ionic liquid (IL) is a molten salt consisting of weakly coordinated cations and anions and displays high ionic conductivity.^21–24^ An IG–MaA gel in an IL exhibited birefringence changes that were 2.7/5.6 times faster than those of a swollen IG–MaA gel in water (Table 1). The properties of the IL, such as its low dielectric constant (<20)^25^ relative to that of pure water (80.4 at 20 °C), accelerated the electrical response of the IG–MaA network.

## Conclusions

Here, the electrical response of IG colloidal dispersions was accelerated by the helical ordering of IG nanotubes and their assembly into a network. Changing the solvent of a colloidal IG dispersion also accelerated its electrical response. Controlling birefringence modulation in an LC system by structuring its components (*i.e.* a mesogen) is important for the design of some devices consisting of an LC system. For example, accelerated birefringence modulation in a colloidal IG dispersion would make it attractive for use in smart, stimuli-responsive active systems such as lighting-control devices for colour filters *via* the further manufacturing of presented IG compositions.

## Conflicts of interest

There are no conflicts to declare.

## Supplementary Material

RA-010-D0RA01092H-s001
