# Supplementary material for: Accelerating the electrical response of solvent-dispersed imogolite nanotubes through structural organisation
Source: RSC Adv. 2020 Mar 5;10(16):9579–81. doi: 10.1039/d0ra01092h (PMC9050123; doi:10.1039/d0ra01092h)
Supplement: RA-010-D0RA01092H-s001 [file RA-010-D0RA01092H-s001.pdf]

Electronic Supplementary Material (ESI) for RSC Advances.
This journal is © The Royal Society of Chemistry 2020

Electronic supplementary information for "Accelerating the electrical response of solvent-dispersed imogolite nanotubes through structural organisation"

Kazuhiro Shikinaka

Table S1 Effect of spacer thickness on the time needed for birefringence (BF) changes after the application or removal of an AC (5 $V_{p\text{-}p}$, 10 KHz). The time was recorded when BF reached a plateau. The samples were then monitored for at least 30 minutes to ensure no additional BF changes occurred.

Sample	Time to BF change after applying AC (s)	Time to BF change after removing AC (s)
Neat IG	85	105
IG + MA (spacer thickness: 30 μm)	17	32
IG + MA (spacer thickness: 584 μm)	52	40

Methods

***Chemicals*:** Deionised water was further purified using a Milli-Q® Advantage A10® system (Millipore™, Eschborn, Germany) and used for all experiments. All chemicals were of reagent grade. With the exception of IG, all chemicals were purchased from Tokyo Kasei Chemicals, Wako Pure Chemical Industries, or Sigma-Aldrich and used as received.

Imogolite (IG) synthesis[S1]

An aqueous solution of $AlCl_3{\cdot}6H_2O$ (Kanto Chem. Co. Inc., Japan) was prepared by dissolving 9.96 g $AlCl_3{\cdot}6H_2O$ in 369 mL purified deionised water. An aqueous solution of Na_4SiO_4 (Junsei Chem. Co. Ltd., Japan) was prepared by dissolving 6.90 g Na_4SiO_4 in 362 mL purified deionised water. The solutions were mixed to obtain a solution containing 12.5 mol L^{-1} Al and 2.5 mol L^{-1} Si. The pH of the mixture was adjusted to 6.0 by rapidly adding ~26 mL of 1.0 mol L^{-1} NaOH (*aq.*). The solution was stirred vigorously for 1 h to avoid local elevations in the pH. The resultant white precipitate was collected *via* centrifugation and resuspended in 400 mL water with stirring. 2.4 L water was added to the suspension, and the solution pH was adjusted to 4.5 by adding 7−8 mL of 1.0 mol L^{-1} HCl. The mixture was carefully and continuously heated and gently stirred for four days at 100 ºC. The as-prepared IG suspension was then cooled to room temperature, and finely powdered sodium chloride (16.4 g) was added with vigorous stirring. The resulting gel was collected *via* centrifugation at 5000 rpm for 30 min and washed with 500 mL water in portions on a 100-nm Millipore filter with suction. Caution was used to prevent the gels from drying. The wet products were added to 1800 mL of stabiliser-free tetrahydrofuran (THF) with stirring. The fluffy precipitate was collected *via* filtration and dried *in vacuo* to obtain a yield of 42%.

Preparation of the aqueous IG dispersions

Measured amounts of IG in pure water were sonicated for 4 h at 150 W (PR-1, Thinky, Japan). Room temperature was maintained by occasionally replacing the water in the sonicator bath. Slightly opaque suspensions of 0.2 mol L^{-1} aluminol functional groups were obtained. The average length of the suspended IG nanotubes was shortened to 131 nm, which was confirmed by examining the nanotubes with a transmission electron microscope.[S1]

Preparation of IG and dicarboxylic acid (IG-DA) gels[S2, S3]

To a 0.16 mol L^{-1} IG water dispersion (1 equivalent with respect to the $–Al(OH)_2$ group), an equal volume of an aqueous maleic acid (MA) or D-malic acid (MaA) solution was added with stirring. Both compounds were generally referred to as carboxylic acid (DA). The mixing ratio was controlled by changing the molar concentration of the DA solution. The concentrations of IG and DA were both 0.08 mol L^{-1}, where $–Al(OH)_2$ and the carboxyl groups were in a 1:1 molar ratio. Gelation was induced by incubating the dispersions for 60 min (MA)[S2] or 20 min (MaA)[S3] at 21 ± 2 °C.

Preparation of an IG-MaA gel dispersed in an ionic liquid[S4]

1-Ethyl-3-methylimidazolium methanesulfonate ([EmIm][$MeSO_3$]) was used as the ionic liquid (IL). To obtain an IG-MaA gel dispersed in the IL, the IL was added to a mixture of IG and DA as described for the IG-DA gels without incubation. The mixture was evaporated for 6 h and dried at 50 ºC for 12 h to obtain a thixotropic IG-MaA gel dispersed in the IL.

Cell preparation for estimation of birefringence changes

Each sample was placed in an estimation cell (Figure S1) for birefringence estimation. After situating the sample between two indium tin oxide (ITO)-coated cover glasses (18 × 18 mm^2; NANOCS Inc., USA, IT25-300-25; ~25 Ohm/sq.), the cover glasses were secured with 30-µm thick double-sided tape (3M Ltd., USA) or 584-µm thick double-sided tape (SEKISUI Ltd., Japan) to form a chamber. To apply the alternating current (AC), two Au wires (80 µm Φ, Tanaka Denshi Kogyo K. K., Japan) were attached to the double-sided tape.

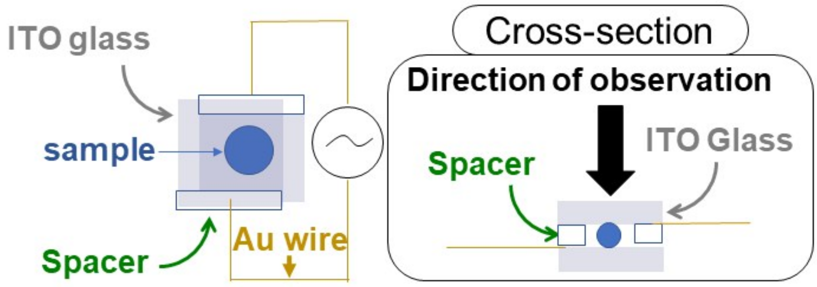

Figure S1. Schematic illustration of the cell assembly for estimating changes in birefringence.

Estimating the electrical response of samples from changes in birefringence

An AC (3−5 V_{p-p}, 10−1000 KHz) was supplied by an AF G1022-type function generator (Tektronix, USA) and applied to the estimation cells. The electrical response of the samples as indicated by changes in birefringence was observed at room temperature using a polarised optical microscope (POM; BX51, Olympus, Japan) under crossed nicols. Images and movies were obtained using a CCD camera (DP74, Olympus, Japan) equipped with image analyser software (Olympus Stream, Olympus).

References

(S1) K. Shikinaka, Y. Koizumi and K. Shigehara, *J. Appl. Polym. Sci.,* 2015, **132,** 41691 (6 pages).

(S2) K. Shikinaka, K. Kaneda, S. Mori, T. Maki, H. Masunaga, Y. Osada and K. Shigehara, *Small,* 2014, **10,** 1813-1820.

(S3) K. Shikinaka, H. Kikuchi, T. Maki, K. Shigehara, H. Masunaga and H. Sato, *Langmuir,* 2016, **32,** 3665-3669.

(S4) K. Shikinaka, N. Taki, K. Kaneda and Y. Tominaga, *Chem. Commun.,* 2017, **53,** 613-616.
